# The Influence of Parent Media Use, Parent Attitude on Media, and Parenting Style on Children’s Media Use

**DOI:** 10.3390/children9010037

**Published:** 2022-01-01

**Authors:** Hye Eun Lee, Ji Young Kim, Changsook Kim

**Affiliations:** 1 Department of Communication & Media, Ewha Womans University, Seoul 03760, Korea; 2 Health and Behavior Studies, Teachers College, Columbia University, New York, NY 10027, USA; jk3565@tc.columbia.edu; 3 Communication & Media Research Center, Ewha Womans University, Seoul 03760, Korea; mulsu@ewha.ac.kr

**Keywords:** children media use, parenting style, parent attitude, parent media use

## Abstract

Parents play a vital role in mediating children’s media use, especially at a young age. We examined the link between the media use of younger children and the media use, attitude toward media, and parenting styles of parents. One thousand and twenty parents of children between 4 and 6 years of age completed a questionnaire on their media use, positive and negative attitudes on media, parenting styles, and the media use of their children. Multigroup structural equation modeling was used to analyze the data. The results showed that there was a significant positive relation between the parent’s media time and the child’s daytime and nighttime media use. Additionally, the parent’s positive attitude toward media use was positively related to the child’s daytime media use, but not the child’s nighttime media use, while the parent’s negative attitude toward media was not associated with the child’s daytime and nighttime media use. Further, among the seven parenting styles, material rewards and autonomy were positively associated with the child’s daytime media use. Discipline was negatively related to the child’s nighttime media use, whereas material rewards were positively associated with the child’s nighttime media use. Collectively, the parent’s positive attitude toward media use was the strongest predictor of the child’s daytime media use, and material rewards were the strongest predictor of the child’s nighttime media use. These results can be of significant use to inform policymakers, researchers, and parents regarding the development of parental guidelines on children’s media use.

## 1. Introduction

Children spend a considerable amount of time consuming media, and this pattern of behavior starts at a young age. Studies have shown that children’s media use is associated with childhood development in areas including physical fitness, psychological well-being, social behavior, and behavioral difficulties [1,2,3,4,5,6]. Parents play a critical role in determining children’s media use since parents spend a sizable portion of time with their children and establish the climate within the household associated with children’s media exposure. Specifically, the parent’s media use, attitudes on media, and parenting styles have been suggested as determinants of children’s media use. Studies have shown that parents with higher screen times also had children with higher screen times [7,8]. The parent’s attitudes and beliefs toward their child’s media use were strong predictors of the amount of time their child spent with screen media [9]. Further, parents with permissive and neglectful parenting styles are likely to allow their children to spend more time on media while parents with authoritarian and authoritative parenting styles are less likely to do so [10,11,12]. Nevertheless, it is unclear which determinant is most strongly associated with children’s media use. Given the significant role of the parents in shaping the child’s media use, identifying the most important parental determinant is vital. The current study aims to examine which parental determinants are associated with young children’s media use and parents’ media use, attitudes on media, and parenting styles.

### 1.1. Children Media Use

Children are increasingly growing up in homes with screen media technologies and are often active users of them. Many children consume more screen media than is recommended by the American Academy of Pediatrics [13] across different devices, such as television, computers, and mobile devices [14]. This phenomenon suggests increased media use among children at a younger age. In fact, in the United States, 70% of children younger than 1 year and 91% of children between 2 and 3 years engage in media use several times per week, despite the recommendation by the American Academy of Pediatrics that children under the age of 2 should spend no time with screen media [14]. Typical 8- to 10-year-old children watch an average of 3 h and 41 min of television and spend over 1 h playing video games daily [15]. In South Korea, children ages 3 through 9 years consume media for 4 h and 45 min each day, which is four times more than what is recommended by the World Health Organization [16]. On average, television, smartphones, tablets, and computers are most frequently used, and the age children begin to use smart devices is decreasing [7]. A cross-sectional study with 350 children ages 6 months through 4 years in the United States showed that 50% of the children had their own television by the age of 4, and 75% had their own mobile device by the age of 4 [17]. Children aged 3 and 4 years used mobile media devices without assistance, and content delivery applications, such as YouTube and Netflix, were popular [16,17]. 

### 1.2. Strengths and Difficulties of Young Children

Children’s media use has implications for strengths and difficulties in childhood development. Several cross-sectional [6] and longitudinal studies [2,5,18] showed positive associations between screen time and behavioral difficulties. Increased media use of children was associated with a decreased level of prosocial behavior [4,6]; however, the level of parent–child interactions mediated the association between children’s media use and level of prosocial behavior [6]. 

Past studies have shown that children’s screen media use relates to their strengths and difficulties as early as the preschool years [5]. Generally, increased use of media at a younger age was linked to reduced physical fitness and psycho-social health [3]. Cross-sectional studies demonstrated that children experiencing excessive screen time also experienced positive associations with poor psychological well-being and behavioral difficulties during preschool [1,6,19]. A longitudinal study with children between 2 and 6 years of age revealed that while the results varied for boys and girls, there was an association between increased media use and poorer well-being outcomes [20]. Another longitudinal study conducted in the Netherlands showed that high television exposure enhanced the risk of preschool children’s behavioral problems [18]. Further, a longitudinal study conducted in South Korea highlighted a positive association between time spent on gaming and voice calls using mobile phones and the risk of developing symptoms of attention deficit disorders [2]. Another study from South Korea, which surveyed mothers and teachers of children who were between the ages of 5 and 6 years demonstrated that increased use of smart devices in children was associated with increased levels of aggression [21]. Collectively, these studies suggested potential adverse effects of excessive media use at a young age. To date, researchers have proposed that underlying mechanisms for these effects include overstimulation of the developing brain and distraction from social and physical activities [22].

### 1.3. Parent’s Media Use and Social Learning Theory

The time that children spend using screen media can be explained using the social learning theory [23]. This theory explains that learning and behaviors result from observing one’s environment. Young children observe their parents, siblings, and surrounding environments and learn from observing the daily routines, interactions and situational response of those people. This includes interactions with screen media within the environment of their household. In this space, parents model media use, and children have a higher chance of mimicking the media use of their parents as a result. For example, a national survey noted that anecdotally, many parents noticed their children mimicking their parents or older siblings by playing with game controllers [14]. Although the survey noted that the children could not use them properly, it supported the theory that children learn to use technologies from observing their environments. Past studies also support the idea of social learning theory by explaining children’s media use in terms of the parent’s media use. Studies have shown that screen time is higher for children with mothers who reported high screen times [7,8]. The researchers suggested that children might use the media use of their mothers as models for their own media use [8]. Thus, we hypothesize:

**H1:**  *A parent’s screen time will be positively related to a child’s media use*.

### 1.4. Parent Attitude and Media

One of the key contributors to a child’s screen time is their parent’s attitude toward media. Past studies have shown that parent attitudes are critical in determining children’s screen time [9,24] along with parent time spent on media technologies and the child’s age [24]. In the United States, parents expressed mixed attitudes toward media. A national study concerning media use of children 6 months to 6 years old consisted of a survey focusing primarily on the role of electronic screen media in children’s lives, including television, videos or DVSs, computers, and video games [14]. Many parents indicated that they encourage their children to spend time with media because they believe that it is beneficial for the children and convenient for the parents, especially when there is a need to keep their children occupied while they finish chores. From an educational standpoint, a similar proportion of parents believed that TV had both positive (38%) and negative effects (31%) on children’s learning, while the majority of the parents expressed that computers helped with learning (70%) and video games hurt learning (49%) [14].

In general, parents who perceive the effects of media positively have children who more often consume media technology. For preschool-aged children, parent attitudes and beliefs regarding children’s media use were strong predictors of the amount of time their children spent with screen media [9]. Similarly, for younger (0–2 years) and older (5–6 years) children, positive parent attitudes toward media were significant predictors of whether their children watched more TV than recommended by the American Academy of Pediatrics [25,26]. Parent attitudes were strongly associated with the use of TV and computers, but less so for mobile devices, such as tablets and smartphones [24]. Overall technology use changed with age, and parent attitudes differentially related to children’s amount of time spent with media for different age groups of children aged between 0 and 8 years old [24]. Parents shape the rules within a household that directly affect the media consumption of younger children. If parents have a positive attitude toward media, they are more likely to have rules that allow media use to foster a positive home environment. If the parents are negative toward media, they are more likely to impose rules that restrict media use. Thus, more positive parent attitudes toward media would likely increase the media consumption of the parents as well as that of their children. We hypothesize:

**H2:**  *Parents with positive attitudes toward media will have a child with higher levels of media use*.

**H3:**  *Parents with negative attitudes toward media will have a child with lower levels of media use*.

### 1.5. Parenting Styles and Media Use

Another factor associated with children’s media use is parenting style. Some researchers have studied parental mediation strategies or practices, which are specific sets of behaviors, while other researchers have studied the overarching parenting dimensions or general context that create the climate for specific parenting practices [27]. Baumrind [28] classified parenting styles into three large categories—authoritarian, authoritative, and permissive—in her classical study, which was later expanded into four different parenting styles based upon levels of demandingness and responsiveness [29]. These four styles include authoritative, authoritarian, indulgent, and neglectful. Authoritative parents are highly demanding and responsive; authoritarian parents are highly demanding but are not responsive; permissive parents are not demanding but highly responsive; and neglectful parents are neither demanding nor responsive [29]. Researchers continued to study the association between parenting styles and child development [28,30,31]. The findings showed that children with authoritative parents had the most favorable developmental outcome, and children with authoritarian and permissive parents were more associated with negative outcomes. Children of neglectful parents had the poorest outcome in terms of psychological competence and academic achievement [31,32,33], psychological outcomes and delinquent behaviors [33,34] and self-efficacy [35].

Different parenting styles can help explain how parents mediate their children’s media use. Parents who exercise lower levels of control over their children (e.g., permissive and neglectful parenting style) are more likely to allow high levels of screen exposure for children between 10 and 11 years of age [10,11]. These parents are more likely to exercise positive parenting and give autonomy to their children. On the other hand, parents who exercise higher levels of control while being supportive (e.g., authoritarian and authoritative parenting styles) are more likely to employ active and restrictive mediation [10,12]. These parents are more likely to utilize monitoring, rules, discipline, harsh punishment, and material rewarding. In terms of children’s media use, children with permissive and neglectful parents are more likely have higher levels of media use, whereas children with authoritarian and authoritative parents would more likely have lower levels of media use.

Additionally, in a study with Dutch parents with young children, it was shown that the parent attitudes regarding the effects of media on children are critical predictors of the parents’ mediation strategies [36]. Parents who believed in the positive influence of media more likely applied supervision, co-use, and active mediation, while parents who were concerned about negative effects were more likely to supervise, restrict, and use technical restrictions on children’s media consumption [36]. In terms of the parenting style, the former parents are more permissive while the latter are more authoritarian. Further, when parents perceived media as a pacifier for the child, they used more restrictive mediations. Parents who believed media to be complicated for their child supervised their child less often, co-used media with their child, and employed technical restrictions more often [36,37,38]. These findings suggest that categorically, parents have a broad perspective regarding the role of media for children, which extends beyond the risk–benefit paradigm [36]. This means that parents not only consider the positive and negative effects of media consumption, but also take the complexity and practical use into account to balance their children’s media use. To better understand the dynamic between parenting style and children’s media use, we hypothesize:

**H4:**  *Children who have parents with permissive and neglectful parenting styles (i.e., exercise positive parenting and give autonomy to children) will have higher levels of media use*.

**H5:**  *Children who have parents with authoritative and authoritarian parenting styles (i.e., monitoring, rules, discipline, harsh punishment, and material rewarding) will have lower levels of media use*.

Given the clear implications of parent media use, parent attitude, and parenting style in the context of children’s media use, as well as children’s strengths and difficulties, the authors of the current study aim to answer the following research question:

RQ1: What factors are the most influential ones among parent’s media use, attitudes on media and parenting styles?

## 2. Materials and Methods

### 2.1. Participants

One thousand and twenty parents of children aged between 4 and 6 years completed a questionnaire between 31 March 2021 and 8 April 2021 through an online survey run by a Korean survey company, MicroEmbrain, which recruited participants from its national panel pool. When the data were collected during the COVID-19 pandemic, kindergartens and preschools were open. Three hundred and forty parents answered the survey for each age group. Fifty percent of the participants were mothers and the other 50% were fathers. Their child’s sex also comprised 50% boys and 50% girls. Finally, 50% of the participants were from double-income families and the other 50% were from single-income families. Sixty-five percent of respondents earned USD 30,000 and more annual household income, and 60.6% held a bachelor’s degree or higher.

### 2.2. Instrument and Measures

The questionnaire was initially constructed in English and then translated into Korean. The equivalence in the process of translation and back-translation was checked by researchers who were fluent in both languages. Along with the main variables, media use (i.e., time spent watching media content using TV, personal device, and smartphone) and demographic information of the children as well as that of the parents was measured. Table 1 shows the reliabilities and descriptive statistics of the variables and correlations among the variables. All the composite variables were computed once their unidimensionality and acceptable reliability were confirmed. Variables were measured with 5-point Likert scales (1 = “strongly disagree” to 5 = “strongly agree”) unless other response formats were listed. All the composite variables were averaged, so the possible range was from 1 to 5.

#### 2.2.1. Children’s Age at First Media Use

Parents were asked to give their child’s age at first media consumption via one item: “How old was your child when he/she first started to watch media content?” The response options were “less than 12 months”, “1-year” “2-year” “3-year” “4-year”, “5-year”, “6-year” and “7-year”.

#### 2.2.2. Child’s Locus of Control Regarding Media Use

The child’s locus of control regarding media use was measured using the modified 5 items from Kendall and Wilcox [39]. An example item is, “My child only watches media content that is scheduled in advance”.

#### 2.2.3. Parents’ Media Time

Each participant was asked to provide the amount of time they spend watching media as well as that of their spouse. The following two questions were used: “How many hours and minutes do you spend watching media on a typical weekday?” and “How many hours and minutes do you spend watching media on a typical weekend?” These were averaged and computed in minutes. 

#### 2.2.4. Parents’ Positive and Negative Attitudes on Media Use

Parents’ degrees of positivity and negativity toward media use were measured based on Elias and Sulkin [40], and Nikken and Jansz [41]. Nine items were asked to measure the positivity of attitudes (e.g., “I think watching media will positively influence my child’s behavioral development”). The negativity of attitudes was measured with two dimensions: intellectual and social. Four items were asked for each dimension. Specifically, “I think watching media will hurt my child’s creativity” for the intellectual dimension, and “I believe watching media will negatively affect my child’s play with friends” for the social dimension. 

#### 2.2.5. Parenting Styles

Parent styles were measured using the Ghent Parental Behavior Scale [42], which was reported to have a solid factor structure in different samples. The original scale has nine dimensions, but two dimensions—inconsistent discipline and ignoring—did not have acceptable reliability in the current study. Accordingly, seven dimensions were further included in the analyses. They are as follows: 11 items for positive parenting (e.g., “I make time to listen to my child, when he/she wants to tell me something”), 4 items for monitoring (e.g., “I keep track of the friends my child is seeing”), 6 items for rules (e.g., “I teach my child to obey rules.”), 4 items for discipline (e.g., “When my child does something that I don’t want him/her to do, I punish him/her”), 4 items for harsh punishment (e.g., “I spank my child when he/she is disobedient or naughty”), 3 items for material rewarding (e.g., “I give my child money or a small present when he/she has done something that I am happy about.”), and 3 items for autonomy (e.g. “I teach my child to solve his/her own problems.”). Due to the obtaining of acceptable reliabilities, the rules dimension had one item (“I teach my child respect for the authorities.”), and the discipline dimension had two items (“When my child has been misbehaving, I give him/her a chore for punishment” and “It happens that I don’t punish my child after he/she has done something that is not allowed”) that were excluded in the analyses. All the dimensions and items are available in the measurement study of Leeuwen and Vermulst [42].

#### 2.2.6. Child’s Media Time

Each participant was asked to click a cell from a 24-h matrix if it corresponded to a time during which their child watches media. The instructions were as follows: “Please check the time box if your child watches media at that time on a typical weekday”, and “Please check the time box if your child watches media at that time on a typical weekend”. If the time was between 7 am and 9 pm, then it was assigned to the child’s daytime media use. If the time was before 7 am or after 9 pm, then it was assigned to the child’s nighttime media use. These were added and computed in hours. The range of the child’s possible daytime media use spanned from 0 to 14, and the range of the child’s possible nighttime media use spanned from 0 to 10. 

### 2.3. Analysis

Structural equation modeling was conducted to test the hypotheses and research question using Mplus 8.0 [43], which uses the maximum likelihood estimation method. To evaluate the model fit, confirmatory fit index (CFI), Tucker–Lewis index (TLI), and root mean square error of approximation (RMSEA) were used. The child’s age at first media use and locus of media regarding media use were controlled in the model since they were related to the main variables. However, the annual household income and education level were not related to the main variables.

## 3. Results

**Figure 1:**
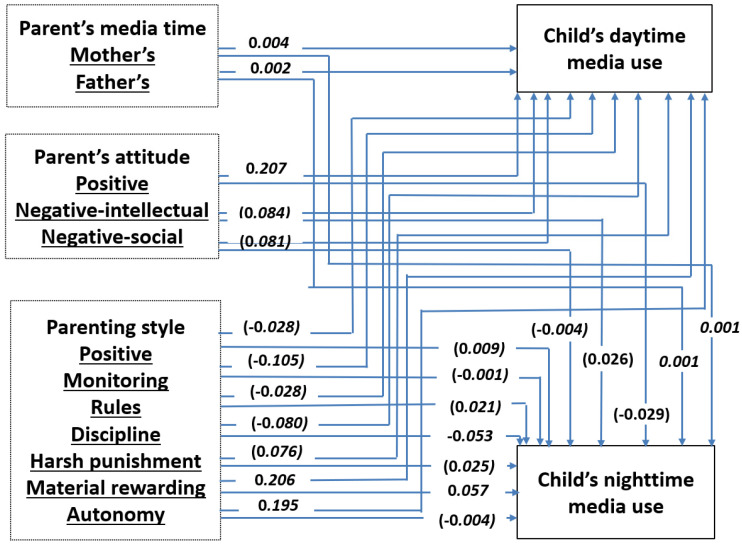
Final model of the relationships among child’s daytime and nighttime media use, parent’s media time, parent’s attitude toward media use, and parenting style. Note that the values are the observed standardized path coefficients. The path coefficients without parentheses are significant at *p* < 0.05.

Acceptable goodness-of-fit indices were obtained for the overall model (χ2(df) = 3277.51(136), *p* < 0.01, CFI = 1.00, TLI = 0.99, and RMSEA = 0.01) [44]. The estimated coefficients are presented in Figure 1. Children’s age at first use and locus of control regarding media use were controlled in the model. *β* is a standardized path coefficient ranging from −1 to 1.

H1 predicted that parents with higher levels of screen time would have children with higher levels of media use. The results supported this hypothesis. The media time of both mother and father showed significant positive effects on both the daytime and nighttime media use of children (*β* = 0.001~0.004, *p* < 0.05).

H2 and H3 hypothesized that the parent’s degrees of positivity and/or negativity on media use would affect the child’s media use. Parents’ positive attitudes toward media use increased child’s daytime media use (*β* = 0.207, *p* < 0.001), but not the child’s nighttime media use (*β* = 0.029, *p* = 272). Parents’ negative attitudes toward media use affected neither the child’s daytime media use nor the child’s nighttime media use. H2 was partially supported; however, the data were not consistent with H3. 

Finally, H4 predicted that parents with permissive and neglectful parenting styles would be positively associated with the child’s media use, while H5 predicted that parents with authoritative and authoritarian parenting styles would be negatively related to the child’s media use. As for parents who practiced material rewarding (*β* = 0.206, *p* < 0.001) and autonomy (*β* = 0.195, *p* < 0.05), the child’s daytime media use increased significantly. The parenting style of discipline (*β* = −0.053, *p* < 0.05) decreased the child’s night time media use, whereas the parenting style of material rewarding (*β* = 0.057. *p* < 0.01) increased the child’s night time media use. The parenting style of autonomy supported H4, and the data from discipline parenting style were consistent with H5. 

RQ1 examined which factors were the most influential among the following: parent’s media use, attitudes toward media, and parenting styles. The results showed that a parent’s positive attitude toward media use is the strongest predictor of the child’s daytime media use, and material rewarding is the strongest predictor of the child’s nighttime media use.

## 4. Discussion

The current study investigated the link between younger children’s media use and parent’s media use, parent attitudes toward media, and parenting styles. The results support that parents play an important role in determining children’s media use. Similar to previous research [7,8], children have higher daytime and nighttime media use when their parents spend more time using media themselves. Further, when parents have a positive attitude toward media, children’s daytime media use increases while children’s nighttime media use does not. However, the parent’s negative attitude toward media does not relate to children’s daytime and nighttime media use. These results are in line with past research that showed that parent attitudes toward children’s media use are strong predictors of the amount of time their children spend with screen media [9]. In terms of parenting styles, children of parents who employ material rewarding and autonomy, among the seven parenting styles, have higher daytime media use. Discipline decreases the child’s nighttime media use, whereas material rewarding increases the child’s nighttime media use. Collectively, the parents’ positive attitude toward media use is the strongest predictor of the child’s daytime media use, and material rewarding is the strongest predictor of the child’s nighttime media use.

Our findings extended past research on the parents’ role in children’s media use and have several implications on the development of parental guidelines on children’s media use. Past studies have identified parental determinants that affect children’s media use—parents’ media use, parent attitudes toward media, and parenting styles—but the current study extends these findings by determining the strongest predictor of children’s media use. It is noteworthy that daytime and nighttime media use are differentiated since media use affects various aspects of children’s lives, including, but not limited to, sleep, brain development, academic performance, nutrition and obesity [45,46,47,48]. There may be differences between daytime and nighttime media use because parents’ general expectations for children’s media use is different during the day than it is at night. During the day, parents may generally be more accepting to increased media use, as children use media for educational purposes or for downtime. Thus, children would use more media if the parents are more positive about media use (i.e., parent’s positive attitude) and allow their children to make decisions on their own (i.e., giving greater autonomy). At night, however, parents may be more against increased media use since media consumption interferes with sleep. This means that parents are more likely to use discipline to mediate children’s media use at night. In general, material rewarding would increase day and nighttime media use as a function of positive reinforcement. In other words, the behavior of media use is more likely to occur in the future when it is followed by reinforcing stimuli, such a praise or reward. Future studies should correlate different time periods and media use to better inform parents on the effects of various times of the day on how children consume media.

For policymakers, these results can aid in the development of specific guidelines to optimize parental support at home, thereby promoting healthy on-screen and off-screen activities. It is important that parents make informed choices given that children are spending more time on media than recommended by public policy. Thus, specific details and guidelines that help parents make these optimal choices should be created based on the findings. The findings have implications for parents as well. The findings support how powerful a role parents can have in shaping their children’s media use [49]. Parents can make informed decisions on how to guide their children’s media use by modifying their own media use and attitude on media. For example, if parents want to decrease their child’s time spent on media, parents could decrease the amount of time they spend on media and use discipline to decrease their child’s nighttime media use. To further extend the findings, future studies should explore the role of parent mediation provided by mothers versus fathers since gender ideologies and stereotypes may be related to children’s screen time [50]. Additionally, the results inform parents on the potential risks of media use for both the parents and children. Only informed parents might change their attitudes, their parenting style, and their own behavior. 

The study is not without limitations. First, the questionnaires were completed by parents through self-reporting, and this method is often biased, due to social desirability bias. This is important to note because public guidelines often advocate limited children’s screen time [51]. Further, only a parent survey with a 24 hr basis scale was used to collect data on children’s media time. Future researchers should collect direct observational data that measure concrete and momentary context to supplement the self-reported data. For example, observational data on parent and children’s time spent on devices would provide more concrete time (e.g., minutes, seconds, and intervals) spent on media. Second, the researchers measured children’s time spent on media without specifying the type of media. Given that past studies indicated different types of media (e.g., gaming and voice calls on mobile phones [2], television [14], and smart devices [7]), future researchers should differentiate the types of media to better understand how the role of parents may differ depending on the type of media. Younger children have guidelines that recommend minimal to no screen time [13]. Thus, different types of media that captures the nuanced use of media at a younger age may be necessary. Third, the current study does not give rise to causal statements. The correlational nature of the analyses describes the parental determinants of children’s media use. It is difficult to rule out the possibility that children may respond differently depending on different genetic predispositions and environmental influences [52]. Fourth, it should be noted that all data were collected during the COVID-19 pandemic. Media use was shown to be higher during the pandemic than before, which may have affected parent media use and attitude toward media. With increased time spent in the household, parents would have spent more time on media and had a more positive attitude toward media consumption for their children, ultimately increasing children’s media use.

Despite the limitations, the current study showed that parental determinants help explain children’s media use. As said previously, different types of media and children’s characteristics could potentially affect the results. Incorporating these variables into future studies would offer researchers more comprehensive insight into the dynamic between parents and children’s media use. This would better inform researchers, policy makers, and parents on building concrete guidelines for children and parents regarding media use.

## Figures and Tables

**Table 1:** Reliabilities, correlations, means, and standard deviations of the main variables.

	1	2	3	4	5	6	7	8	9	10	11	12	13	14	15	16
Child’s age at first use	-															
2. Child’s locus of control	0.12 **	(0.76)														
3. Mother’s media time	0.03	−0.06 *	-													
4. Father’s media time	0.03	−0.12 **	0.58 *	-												
5. Positive attitude on media use	0.10 **	0.29 **	0.11 **	0.08 *	(0.89)											
6. Negative attitude on media use -intellectual	−0.03	−0.05	−0.04	−0.02	−0.22 **	(0.84)										
7. Negative attitude on media use -social	0.01	−0.13 **	−0.02	0.04	−0.15 **	0.61 **	(0.84)									
8. Positive parenting	−0.07 *	0.22 **	−0.06 *	−0.09 **	0.13 **	−0.14 **	−0.16 **	(0.92)								
9. Monitoring	0.02	0.18 **	−0.09 **	−0.06	0.09 **	−0.06	−0.02	0.48 **	(0.74)							
10. Rules	−0.09 **	0.06	−0.05	−0.07 *	0.03	−0.09 **	−0.05	0.57 **	0.46 **	(0.92)						
11. Discipline	−0.04	0.04	0.03	0.07 *	0.11 **	0.08 *	0.17 **	0.00	0.12 **	0.19 **	(0.77)					
12. Harsh punishment	0.07 *	0.00	0.08 **	0.11 **	0.08 **	0.20 **	0.22 **	−0.40 **	−0.12 **	−0.25 **	0.32 **	(0.90)				
13. Material rewarding	−0.02	0.09 **	0.01	0.03	0.24 **	0.11 **	0.20 **	−0.10 **	−0.03	−0.09 **	0.20 **	0.25 **	(0.71)			
14. Autonomy	0.00	0.21 **	−0.06 *	−0.06 *	0.12 **	−0.11 **	−0.12 **	0.42 **	0.28 **	0.42 **	0.08 *	−0.19 **	0.04	(0.82)		
15. Child’s daytime media use	−0.11 **	−0.22 **	0.32 **	0.27 **	0.06 *	0.06	0.10 **	−0.08 **	−0.10 **	−0.03	0.03	0.10 **	0.13 **	−0.01	-	
16. Child’s nighttime media use	−0.04	−0.13 **	0.15 **	0.16 **	0.03	0.04	0.05	−0.05	−0.04	−0.03	−0.03	0.06 *	0.10 **	−0.04	0.08 *	-
Mean	2.89	3.06	127	128	3.06	2.89	2.83	4.08	3.81	4.28	3.37	1.81	2.80	3.82	2.41	0.18
Standard deviation	1.14	0.65	94.55	87.35	0.65	0.73	0.78	0.50	0.66	0.53	0.69	0.84	0.78	0.54	1.53	0.43
Range	1–7	1–5	0–630	0–630	1–5	1–5	1–5	2.18–5	1–5	2.5–5	1–5	1–4.75	1–5	2–5	0–13	0–3.71

Note 1. ** *p* < 0.001, * *p* < 0.05. Note 2. Reliabilities are reported in parentheses on the diagonal.

## Data Availability

Qualified researchers can obtain the data from the corresponding author (hyeeun-lee77@ewha.ac.kr). The data are not publicly available due to privacy concerns imposed by the IRB.
